# Presence of a cryptic *Onchocerca* species in black flies of northern California, USA

**DOI:** 10.1186/s13071-021-04990-1

**Published:** 2021-09-15

**Authors:** Matthew Kulpa, Kimberly J. Nelson, Alana M. Morales, Bonnie M. Ryan, Michelle L. Koschik, Jamesina J. Scott, Guilherme G. Verocai

**Affiliations:** 1 Department of Veterinary Pathobiology, College of Veterinary Medicine and Biomedical Sciences, Texas A&M University, College Station, TX 77843 USA; 2 San Gabriel Valley Mosquito and Vector Control District, West Covina, CA USA; 3 Department of Infectious Diseases, College of Veterinary Medicine, University of Georgia, 501 D.W. Brooks Drive, Athens, GA 30602 USA; 4 Lake County Vector Control District, 410 Esplanade St., Lakeport, CA 95453 USA

**Keywords:** Cervidae, Filarial parasites, Filarioidea, Onchocerciasis, Parasite biodiversity, Vector-borne diseases, Xenomonitoring

## Abstract

**Background:**

Black flies (Diptera: Simuliidae) serve as arthropod vectors for various species of *Onchocerca* (Nematoda: Onchocercidae) that may be associated with disease in humans, domestic animals, and wildlife. The emergence of zoonotic *Onchocerca lupi* in North America and reports of cervid-associated zoonotic onchocerciasis by *Onchocerca jakutensis* highlight the need for increased entomological surveillance. In addition, there is mounting evidence that *Onchocerca* diversity in North America is far greater than previously thought, currently regarded as *Onchocerca cervipedis* species complex. This study reports new geographic records and black fly vector associations of an uncharacterized *Onchocerca* species.

**Methods:**

To better understand the biodiversity and geographic distribution of *Onchocerca*, 485 female black flies (2015: 150, 2016: 335) were collected using CO_2_-baited traps from February to October 2015–2016 in Lake County, northern California, USA. Individual flies were morphologically identified and pooled (≤ 10 individuals) by species, collection date, and trap location. Black fly pools were processed for DNA extraction, and subsequent PCR and sequencing targeting of the NADH dehydrogenase subunit 5 gene of filarioids.

**Results:**

Among the pools of black flies, there were 158 individuals of *Simulium tescorum* (2015: 57, 2016: 101), 302 individuals of *Simulium vittatum* (*sensu lato* [s.l.]) (2015: 82, 2016: 220), 16 individuals of *Simulium clarum* “black” phenotype (2015: 5, 2016: 11), and 13 individuals of *S. clarum* “orange” phenotype (2015: 6, 2016: 7). PCR analysis revealed the percentage of filarioid-positive pools were 7.50% (*n* = 3) for *S. tescorum*, 3.75% (*n* = 3) for *S. vittatum* (s.l., likely *S. tribulatum*), 7.69% (*n* = 1) for *S. clarum* “black” phenotype, and no positives for *S. clarum* “orange” phenotype. Genetic distance and phylogenetic analyses suggest that the northern California *Onchocerca* isolates belong to the same species reported in black flies from southern California (average pairwise comparison: 0.32%), and seem closely related to *Onchocerca* isolates of white-tailed deer from upstate New York (average pairwise comparison: 2.31%).

**Conclusion:**

A cryptic *Onchocerca* species was found in Lake County, California, and may be a part of a larger, continentally distributed species complex rather than a single described species of North America. In addition, there are at least three putative vectors of black flies (*S. clarum*, *S. tescorum*, *S. vittatum*) associated with this cryptic *Onchocerca* species. A comprehensive reassessment of North American *Onchocerca* biodiversity, host, and geographic range is necessary.

**Graphical abstract:**

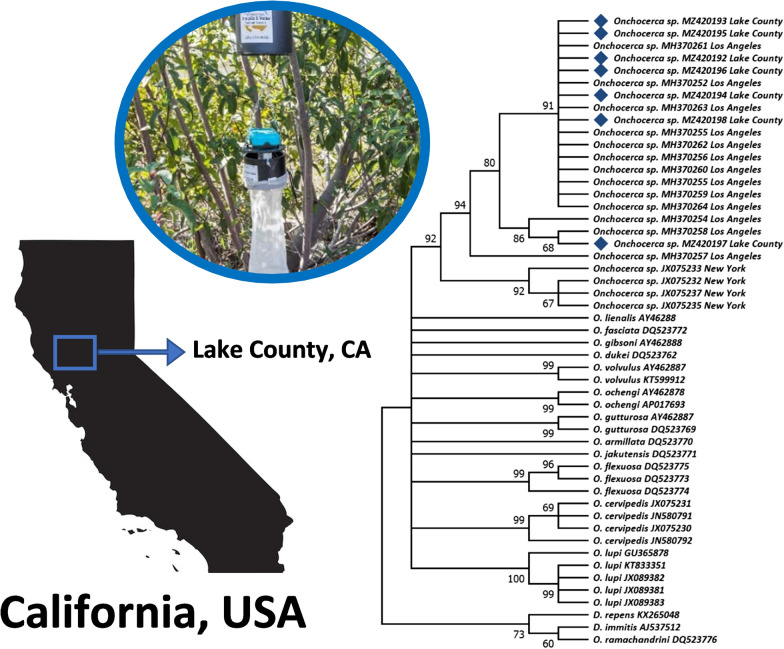

## Background

*Onchocerca* Diesing, 1841, a genus of filarial nematodes, is a globally distributed, vector-borne parasite that infects a wide variety of species that includes both animals and humans [1]. Well-known species of *Onchocerca* include *Onchocerca volvulus* (Leuckart, 1893), also known as the agent of river blindness in humans, and the zoonotic parasite *Onchocerca lupi* Rodonaja, 1967, the agent for causing canine ocular onchocerciasis [2]. *Onchocerca* species are transmitted via blood-sucking dipteran vectors, including black flies (Simuliidae) and biting midges (Ceratopogonidae), to definitive mammalian hosts [1].

Despite the zoonotic potential and possible deleterious impacts to host health of most *Onchocerca* species, little is known about the clinical and ecological significance of the ungulate parasite *Onchocerca cervipedis* Wehr and Dikmans, 1935, or what is commonly known as the “foot worm.” Described nearly a century ago [3], *O. cervipedis* has an extensive distribution range from areas of Central America to Canada, and infects a variety of cervids including the white-tailed deer *Odocoileus virginianus* (Zimmermann, 1780); mule deer *Odocoileus hemionus* (Rafinesque, 1817); moose *Alces americanus* Clinton, 1822; elk or wapiti *Cervus canadensis* Erxleben, 1777; and caribou *Rangifer tarandus* (Linnaeus, 1758); and the antilocaprid pronghorn *Antilocapra americana* (Ord, 1815) [4–16]. *Onchocerca cervipedis* has always been assumed to be the only *Onchocerca* species to infect these North American ungulates; however, there is mounting evidence that suggests otherwise. Recent studies have shown that *Onchocerca* isolates from the skin of white-tailed deer from New York [17] were genetically distinct from isolates of moose from northern Canada [15]. In addition, cryptic *Onchocerca* DNA was discovered from black fly vectors of southern California, and blood analysis supports the notion of a possible Cervidae host [18]. Therefore, all previous reports on *Onchocerca* across the Americas, including ungulate host and vector associations, require a comprehensive re-evaluation [15, 17, 18].

In order to shed further light on the cryptic diversity of species within *Onchocerca* from North America, we molecularly screened putative black fly vectors trapped in Lake County, NC, USA, for filarial nematode DNA. We discuss these results in the current context of known cryptic biodiversity and historical biogeography of *Onchocerca* in North America.

## Methods

### Black fly collection

**Fig. 1:**
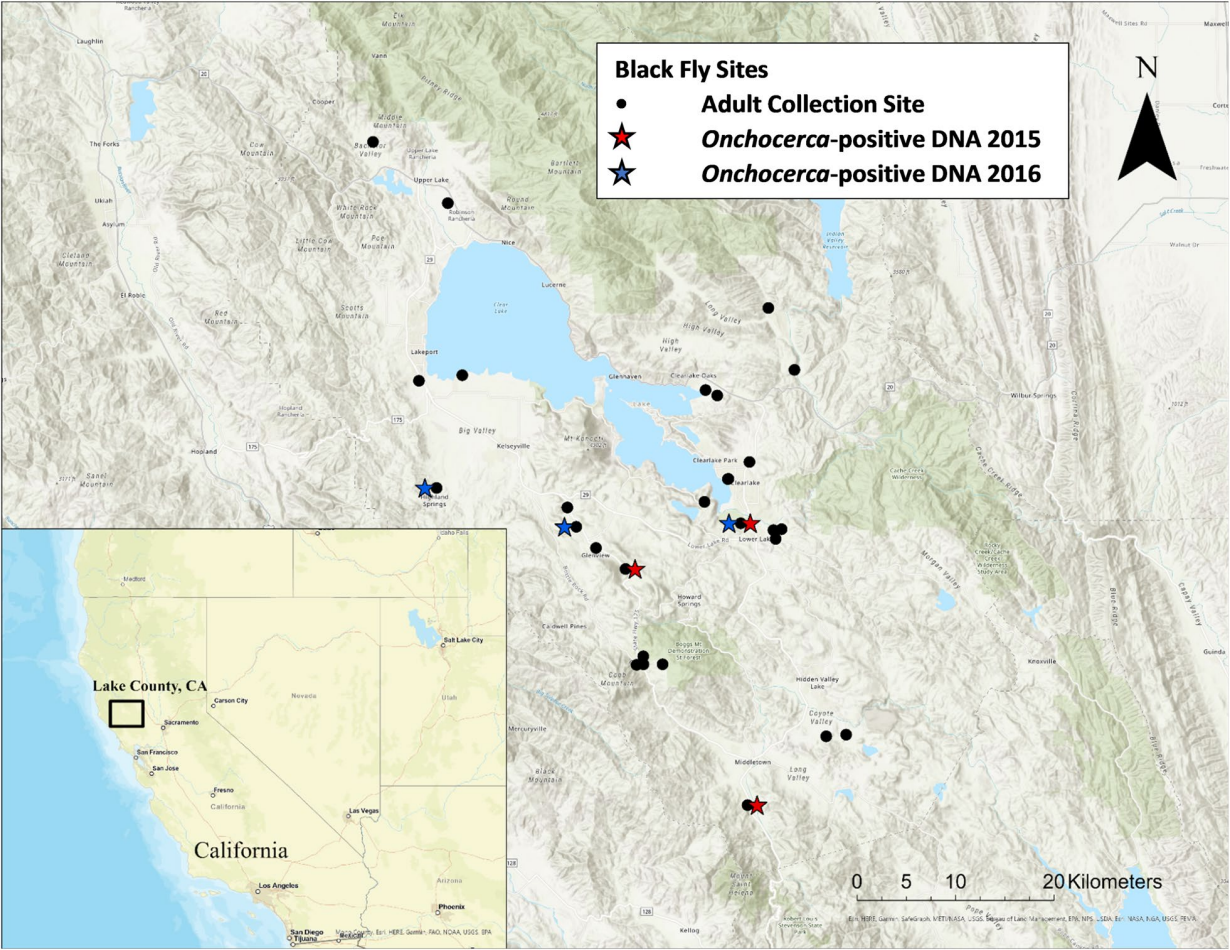
Locations of adult black fly collection sites in Lake County, California. Each collection site is marked with a black dot. Sites denoted with a right-staggered red star indicate an *Onchocerca*-positive PCR test in 2015 (*n* = 4), and sites denoted with a left-staggered blue star indicate an *Onchocerca*-positive PCR test in 2016 (*n* = 3)

Lake County, California, was the designated area targeted for black fly collection. Lake County is located in one of the broad valleys of northern California (122°50′ W, 39°00′N) and contains the largest freshwater lake entirely in California, Clear Lake [19]. Through coordination with the Lake County Vector Control District, female black flies were caught by CDC-style miniature CO_2_-baited mosquito traps (John W. Hock Company, Gainesville, FL, USA). Dry ice kept in a cooler served as source of CO_2_, and traps were set overnight at various locations around the shores of Clear Lake, weekly or biweekly, between April 2015 and October 2016 (Fig. 1). Once collected, the black flies were morphologically identified to species/species-complex level according to taxonomic keys [20]. Adult *S. clarum* black flies were recognized by a distinct three-striped scutal pattern, but were differentiated by stripe color type. All samples were stored at −80 °C until further analysis.

### Molecular screening and sequencing

Individual flies were morphologically identified and pooled (≤ 10 individuals) by species, collection date, and trap location (Table 1; Fig. 1). DNA extraction of pools of black flies was performed manually using the Qiagen DNeasy^©^ Blood and Tissue Kit (Qiagen, Valencia, CA, USA). Briefly, black flies were macerated with sterile plastic pestles in an Eppendorf tube, and homogenized with ATL buffer and proteinase K. Samples were then incubated in a dry heat block for 45 min at 56 °C, and then centrifuged for 5 min at 8000×*g*. The remaining protocol steps followed the manufacturer’s instructions. DNA lysates were kept refrigerated at −20 °C until further processing.

**Table 1:** Summary of positive black flies according to their year and collection sites

	Number examined	Number of pools			Positive black fly pools	Coordinates	Location	Percentage of positive pools by species (%)	
2015									
* S. clarum* “black”	5	3			(1) SCB-15-039	38°53′21.9′′N, 122°43′53.6′′W	Kelseyville	33.3	
* S. clarum* “orange”	6	6			None	–	–	0.0	
* S. tescorum*	57	17			(1) ST-15-010	38°43′16.7′′N, 122°37′12.8′′W	Middletown	5.9	
* S. vittatum*	82	31			(1) SV-15-020A	38°55′3.8′′N, 122°35′20.9′′W	Lower Lake	6.5	
					(2) SV-15-043	38°55′3.8′′N, 122°35′20.9′′W	Lower Lake		
Total	150	57			4			7.0	
2016									
* S. clarum* “black”	11	10			None	–	–	–	
* S. clarum* “orange”	7	7			None	–	–	–	
* S. tescorum*	101	23			(1) ST-16-011	38°56′49.5′′N, 122°54′14.3′′W	Lakeport	8.7	
					(2) ST-16-014	38°55′10.2′′N, 122°46′35.5′′W	Kelseyville		
* S. vittatum*	220	49			(1) SV-16-030A	38°55′19.1′′N, 122°37′35.0′′W	Lower Lake	2.0	
Total	335	89			3			3.4	
2015–2016									
* S. clarum* “black”	16	13			1	–	–	7.7	
* S. clarum* “orange”	13	13			0	–	–	0.0	
* S. tescorum*	158	40			3	–	–	7.5	
* S. vittatum*	302	80			3	–	–	3.8	
Overall total	485	146			7			4.8	

Black flies were collected in the 2015–2016 field season using CO_2_ -baited traps in the Lake County, California area. Four species of black flies were caught: *S. clarum* (black); *S. clarum* (orange) *S. tescorum*; and *S. vittatum*. However, *S. clarum* (orange) had no positive individuals. Each row denotes the number of black flies examined, the number of pools (*n* =  ≤ 10), the positive black fly pools, coordinates and cities of where the positive was located, and the percentage of positive pools by species

Polymerase chain reactions (PCR) targeting the mitochondrial NADH dehydrogenase subunit 5 (*nd5*) gene of filarioid nematodes, using the primers ND5-Ov5A-F (5′-TTGGTTGCCTAAGGCTATGG-3′) and ND5OvC-R (5′-CCCCTAGTAAACAACAAACCACA-3′) [21]. Cycling conditions consisted of 95 °C for 2 min, followed by 35 cycles of 95 °C for 30 s, 50 °C for 45 s, and 72 °C for 30 s, and a final extension at 72 °C for 5 min, following previously published protocols [18].

Potential PCR products were subjected to agarose gel to determine if amplicon was present. An E.Z.N.A. Cycle Pure Kit (Omega Bio-tek, Norcross, GA, USA) was used to purify DNA using the manufacturer’s protocol. Products were then directly sequenced with the same primers using the BigDye Terminator Cycle Sequencing Kit.

### Phylogenetic analysis

Sequences were aligned and edited using MEGA X software [22]. Phylogenetic trees of the partial *nd5* gene (427 bp) were constructed by utilizing the maximum likelihood method and Tamura-Nei model with gamma distribution in 2000 bootstrap replicates. All sequences at the *nd5* gene for *Onchocerca* species available through GenBank were included. *Dirofilaria immitis* (Leidy, 1856) and *Dirofilaria repens* Railliet and Henry, 1911 were used as outgroups within the family Onchocercidae.

### Taxonomy of simuliid vectors and mammalian hosts for *Onchocerca*

The taxonomy of black flies and artiodactyl mammalian hosts followed the most recent and comprehensive literature [20, 23, 24].

## Results

A total of 485 black flies were collected from 27 different collection sites in the Lake County area (Fig. 1). Overall, 150 flies were collected in 2015, and 335 flies in 2016, representing three black fly species. Of these, 158 individuals were identified as *Simulium tescorum* Stone and Boreham, 1965 (2015: 57, 2016: 101), 302 individuals of *Simulium vittatum* Lugger, 1897 (sensu lato [s.l.], likely *S. tribulatum*) (2015: 82, 2016: 220), 16 individuals of *Simulium clarum* (Dyar and Shannon, 1927) “black” phenotype (2015: 5, 2016: 11), and 13 individuals of *Simulium clarum* “orange” phenotype (2015: 6, 2016: 7).

Regarding the samples collected in 2015, a total of 2/31 *S. vittatum* pools (6.5%), 1/17 *S. tescorum* pools (5.9%), and 1/3 *S. clarum* “black” phenotype pools (33.3%) were positive for filarioid DNA and subsequently sequenced for *Onchocerca* DNA (Table 1). In 2016, a total of 1/49 *S. vittatum* pools (2.0%) and 2/23 *S. tescorum* pools (8.7%), were positive for filarioid DNA and subsequently sequenced (Table 1). All positive *S. vittatum* (*n* = 3) pools came from Lower Lake, while each of the *S. tescorum* positive pools (*n* = 3) came from three different locations: Middletown, Lakeport, and Kelseyville. The single positive *S. clarum* “black” phenotype (*n* = 1) was also found in Kelseyville (Table 1).

**Fig. 2:**
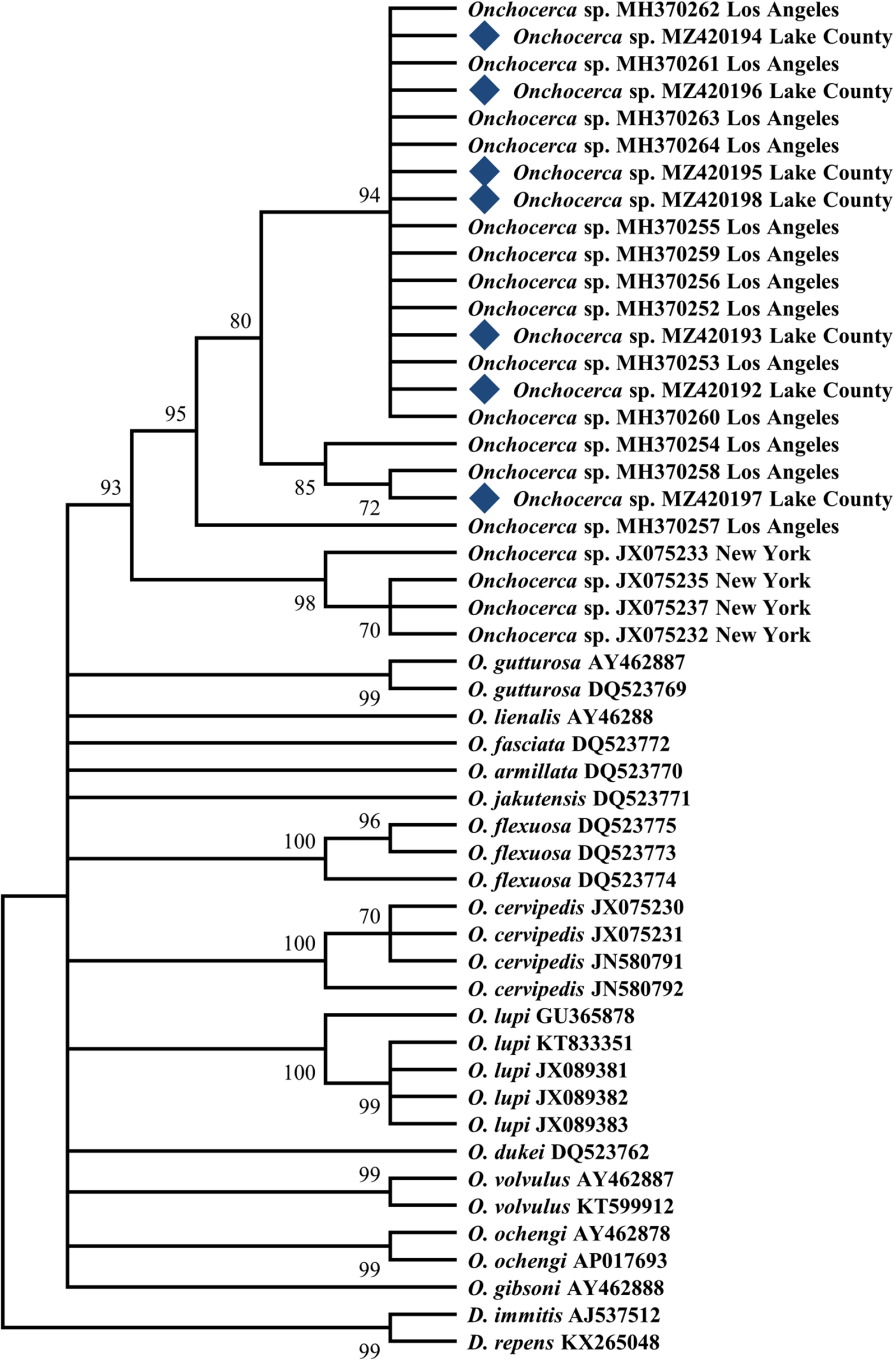
Maximum likelihood tree depicting phylogenetic relationship of the *nd5* gene between species of known *Onchocerca* and the cryptic *Onchocerca* DNA found across geographic isolates of *Onchocerca* in California and New York, USA, created with MEGA X. Branches with less than 50% bootstrap were collapsed and bootstrap support shown besides branches indicate 2000 replicates. All cryptic DNA samples obtained from black flies from Lake County, California, are denoted with a black diamond and have been accessioned in GenBank (MZ420192; MZ420193; MZ420194; MZ420195; MZ420196; MZ420197; MZ420198)

All seven generated *nd5* sequences were deposited in the GenBank (Accession numbers: MZ420192-98). Phylogenetic analysis showed strong support that the Lake County *Onchocerca* isolates in northern California are conspecific with the isolates from Los Angeles in southern California (94% bootstrap support), and likely belong to an uncharacterized species (Fig. 2). In addition, the upstate New York *Onchocerca* isolates appear to be closely related to both Californian isolates (92% bootstrap support) (Fig. 2). Other *Onchocerca* isolates or species that have been reported from North American wildlife, namely *O. cervipedis* sensu Verocai et al. [15] of moose from Canada, and *O. lupi* reported from companion animals, coyotes, and humans in North America [25–27], were not included within this clade.

**Fig. 3:**
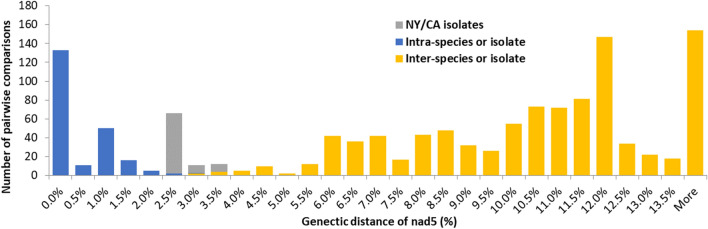
The number of base substitutions per site are calculated and the evolutionary divergence is estimated between sequences. Each bar represents the total amount of pairwise comparisons of the *nd5* gene, or nucleotide sequence divergence, from 50 different *Onchocerca* species or isolates. Evolutionary analysis was done using MEGA X and a Tamura-Nei model with gamma distribution. Blue bars indicate supposed intra-isolate comparisons and orange bars indicate supposed inter-isolate comparisons of all *Onchocerca* species or discovered isolates. Lake County, CA and Los Angeles, CA isolate comparisons have been treated as intra-specific species. Gray bars indicate NY-CA isolate comparisons

Pairwise distance data (Table 2) also show strong support for each of the three geographic isolates being closely related to one another. Of the three, both Californian isolates are more similar to each other, with a pairwise distance averaging 0.32% (0.00–2.54%). The New York *Onchocerca* isolate had an average pairwise distance of 2.31% (2.12–3.27%) when compared to the Lake County isolates, and 2.34% (2.12–3.27%) when compared to the Los Angeles isolates. On the other hand, when Lake County isolates were compared to *O. cervipedis* sensu Verocai et al. [15] isolates, there was a pairwise distance of 10.04% (9.64–10.64%). These genetic distances are similar to interspecific *Onchocerca* species comparisons like *O. lupi*, 11.75% average (11.24–11.86%), rather than intraspecific comparisons (Table 2; Fig. 3). The majority of pairwise comparisons fall outside the range of ~ 2.00–5.00% (Table 2; Fig. 3), which is comparable to other studies comparing interspecific versus intraspecific based on pairwise distances at the partial *cox-1* gene of the genus *Onchocerca* [2]. However, when the New York isolate is compared to either Californian isolate, all pairwise comparisons fall within the range of ~ 2.00–5.00%. While evidence clearly indicates that all Californian isolates are conspecifics (Table 2; Fig. 3), the phylogenetic relationships among the New York and Californian isolates remain ambiguous. Table 3 shows the average and range percent identity among Lake County *Onchocerca* isolates and other isolates also shown in Table 2 using BLAST analysis.

**Table 2:** Average pairwise comparisons, with ranges in parentheses, of *nd5* gene with different *Onchocerca* isolates or species

Onchocerca isolate	Lake County, CA	Los Angeles, CA	Upstate New York	*Onchocerca* sp.	*Onchocerca lupi*	Reference
Lake County, CA	0.24% (0.00–0.95%)					Present study
Los Angeles, CA	0.32% (0.00–2.26%)	0.48% (0.00–2.54%)				[18]
Upstate New York	2.31% (2.12–3.27%)	2.34% (2.12–3.27%)	0.24% (0.00–0.48%)			[17]
*Onchocerca* sp.	10.04% (9.64–10.64%)	9.93% (7.65–11.11%)	9.47% (8.61–9.77%)	0.12% (0.00–0.24%)		[15]
Onchocerca lupi*	11.75% (11.24–11.86%)	11.82% (11.24–12.21%)	10.30% (9.18–10.70%)	10.99% (10.53–11.13%)	0.61% (0.00–1.51%)	Various sources

*Onchocerca* isolates are broken down by region (Lake County, CA; Los Angeles, CA; and Ithaca, NY) or by the species it is from (*O. lupi; Onchocerca* sp.). *Onchocerca lupi* was chosen because it is a North American *Onchocerca* species that is not considered part of the hypothesized *Onchocerca cervipedis* species complex

**Table 3:** Average percent identity of Lake County isolates compared to other known *Onchocerca* isolates, using NCBI BLAST analysis, at the *nd5* gene level

	Lake County, CA						
Accession numbers	MZ420192	MZ420193	MZ420194	MZ420195	MZ420198	MZ420196	MZ420197
Los Angeles, CA [18]	99.76% (98.57–100%)	99.76% (98.57–100%)	99.76% (98.57–100%)	99.76% (98.57–100%)	99.76% (98.57–100%)	99.76% (98.57–100%)	99.26% (97.87–100%)
Upstate New York [17]	96.42% (94.57–100%)	96.42% (94.57–100%)	96.42% (94.57–100%)	96.42% (94.57–100%)	96.42% (94.57–100%)	96.42% (94.57–100%)	95.70% (94.03–97.37%)
*Onchocerca* sp. [15]	92.09% (91.94–92.04%)	92.08% (91.92–92.38%)	92.09% (91.94–92.04%)	92.09% 91.94–92.04%)	92.08% (91.92–92.38%)	92.12% (91.86–92.43%)	91.95% (91.63–92.36%)
*Onchocerca lupi*	91.81% (91.74–92.00%)	91.79% (91.72–92.00%)	91.81% (91.74–92.00%)	91.81% (91.94–92.00%)	91.79% (91.72–92.00%)	91.82% (91.76–92.00%)	92.05% (91.99–92.24%)

*Onchocerca* isolates are broken down by region (Lake County, CA; Los Angeles, CA; and Ithaca, NY) or by the species it is from (*O. lupi*; *Onchocerca* sp.).* Onchocerca lupi* was chosen because it is a North American* Onchocerca* species that is not considered part of the hypothesized *Onchocerca cervipedis* species complex

## Discussion

Our study identified cryptic *Onchocerca* DNA in three different *Simulium* species in southern California, USA. We discovered that *Onchocerca* isolates found in black flies in Lake County, northern California, belong to the same cryptic *Onchocerca* species previously found in black flies in Los Angeles County, southern California [18]. Corroborating the findings from southern California, *Onchocerca* DNA was detected in two black fly species: *S. vittatum* (s.l.) and *S. tescorum* [18] (Table 1). In addition, a third species of black fly was shown to carry the same cryptic *Onchocerca* DNA: *S. clarum* belonging to the “black” phenotype (Table 1).

Phylogenetic analyses of the *nd*5 gene demonstrate that the cryptic *Onchocerca* found in southern and northern California black flies (present study; [18]) and the equally cryptic *Onchocerca* isolate found in New York, northeastern USA [17] represent one individual clade with little genetic divergence (Fig. 2). However, a definitive conclusion on whether the Californian isolates are conspecific with the New York isolates cannot yet be determined (Table 2; Fig. 3). Further studies targeting a multilocus approach could help shed light on the exact phylogenetic relationships and taxonomic status of these geographically distant isolates. This notion is best exemplified by comparing the *nd5* gene to the *cox-1* gene, which appears to exhibit greater diversity within the cryptic *Onchocerca* isolates [18]. In addition, at this stage, it is not possible to conclude that the cryptic species present in northern California belongs to the originally described *O. cervipedis.* In the original description of the species by Wehr and Dikmans [3], the authors used specimens from two different locations and at least two different hosts, including *O. virginianus* and *O. hemionus* from Montana, USA, and *O. hemionus* from British Columbia, Canada. To further elucidate this taxonomic conundrum, isolates from these hosts and locations should be collected, morphologically re-evaluated, molecularly characterized, and subsequently compared to these many isolates within the *Onchocerca* complex.

### Molecular screening and putative vectors of cryptic *Onchocerca* isolates

The finding of cryptic *Onchocerca* DNA through molecular screening of arthropod vectors (i.e., xenomonitoring) provides a straightforward approach to understanding more about parasite biodiversity, geographic distribution, and putative vector associations. Moreover, the utilization of xenomonitoring of North American parasites allows for concurrent monitoring of other similar *Onchocerca* species, such as the zoonotic *O. lupi*, that are of current public health concern [28]. However, despite these advantages, implication of a given arthropod species in the transmission of *Onchocerca* should be cautiously interpreted until further demonstrated by recovering infective third-stage larvae or parasite DNA from the head of the vector, and/or experimentally. Comparable to Verocai et al. [18], our results showed that the positivity rate for *Onchocerca* DNA was low in the black fly populations. This is similar to other filarial nematode studies that revealed low positive prevalence rates of *O. lupi* in southern California [28], *O. volvulus* in Africa [29, 30], and *Wuchereria bancrofti* (Cobbold, 1877) in American Samoa and Guinea [31–33].

Our study also provided evidence for an additional species of black fly as a probable vector of this *Onchocerca* species. Although three black fly species have been implicated as possible intermediate hosts for this *Onchocerca*, it should be noted that the CO_2_ trapping method utilized may impact the abundance and species composition of black flies caught [34]. According to the literature, *S. clarum* has been reported to feed on a variety of mammals (horses, cattle, rabbits, and humans) and birds [35, 36]. The finding of DNA of an *Onchocerca* species possibly associated with a cervid host(s) suggests that these mammals may serve as a blood source for this dipteran, similar to that of *S. tescorum* and *S. vittatum*, as suggested by Verocai et al. [18, 20]. However, *S. clarum* is restricted to the California Central Valley region near the present study site of Lake County [20]. Similarly, *S. tescorum* has been reported with a limited range, spanning only California and Arizona [20, 23]. This means that even if these two vectors are competent hosts for this *Onchocerca* species, they would only contribute to the transmission within their more restricted distribution. In contrast, species within the *S. vittatum* complex, which includes *S. tribulatum*, have a widespread distribution across North America, including both California and New York [23].

### Definitive hosts of cryptic *Onchocerca* isolates

While relevant literature suggests that this *Onchocerca* isolate is associated with cervid hosts [17, 18], there is a lack of experimental data to definitively confirm this hypothesis. However, the recent discoveries of at least two or more genetic *Onchocerca* isolates in North America hypothesized to be associated with at least three of the cervid hosts (i.e., mule deer, white-tailed deer, and moose) raise many questions regarding *Onchocerca*–host assemblages. Of these three cervid hosts, only the mule deer’s range encompasses southern California, including Los Angeles County [37–39]. Thus, it was reasonably hypothesized that the mule deer could be the putative host to the *Onchocerca* isolate from southern California if the parasite is truly associated with cervid hosts [18]. Lake County also includes the range of the mule deer [37]; however, unlike southern California, Lake County is also home to the Californian tule elk, or *Cervus elaphus nannodes* Merriam, 1905 [40]. This elk subspecies was hunted to near extinction in the late 1800s, and now has a thriving population in California. According to most recent data, about 6000 tule elk populate California, including many herds that live near the Lake County region of northern California where black flies were sampled for this current study [40–42]. While there was no blood meal analysis completed, it is possible that these cervids could be a blood meal source for black flies and consequently be a potential host to the hypothesized *O. cervipedis* species complex [8]. Ideally, adult worms or microfilariae should be sampled from necropsied elk hosts and molecularly analyzed to confirm its definitive host status.

Species within *O. cervipedis* complex have been reported in a variety of locations across North America in the six ungulate hosts: pronghorn from Idaho [9]; moose from Alaska, Alberta, British Columbia, and the Northwest Territories [12, 14–16, 43]; elk from Montana [8]; mule deer from Arizona, California, Montana, Utah, Wyoming, and British Columbia [3–5, 7, 8, 10, 18, 44–52]; white-tailed deer from Arizona, Missouri, Montana, New York, Oregon, Pennsylvania, and British Columbia, and also from Costa Rica [3, 5, 6, 8, 13, 17, 46, 50, 53–57]; and caribou from Alaska and British Columbia [11, 15]. Additional records from *Odocoileus* from Colorado, Idaho, and Montana were reported as “deer,” without species designation [58–62]. Therefore, it can be inferred that sample collection should begin in these reported locations and include all six ungulate hosts when obtaining biological samples. Recovery of nematodes from necropsy, with subsequent morphological and DNA identification, will confirm parasitic infection of a definitive host and aid in interpreting the distribution of cryptic *Onchocerca* isolates.

### Evolutionary history and ecological considerations of cryptic *Onchocerca* isolates

Currently, it is hypothesized that the two, and possibly more, known *Onchocerca* species (i.e., *O. cervipedis* sensu Verocai et al. [15] and the clade comprising the Californian and New York isolates [17, 18]) are the result of independent expansion events from Palearctic ungulates hosts colonizing from across the Bering Land Bridge into the Nearctic [63–65]. It is currently unknown if the finding of at least two *Onchocerca* species is the result of a small, incomplete sampling of larger species diversity or the true representation of diversity in North America. Nevertheless, there is substantial evidence from eastern Asia for prior underestimation of *Onchocerca* species diversity and richness. For instance, *Onchocerca suzukii* Yagi, Bain and Shoho, 1994, *Onchocerca eberhardi* Uni et al., 2007, and *Onchocerca takaokai* Uni, Fukuda and Bain, 2015, have been recently described from wild ungulates of Japan [66–68]. Furthermore, *Onchocerca borneensis* Uni, Mat Udin and Takaoka, 2020 [69], was described in bearded pigs of Borneo with additional molecular evidence suggesting that two closely related parasites, *Onchocerca dewittei* Bain, Ramachandran, Petter and Mak, 1977, and *Onchocerca japonica* Uni, Bain and Takaoka, 2001*,* which were considered subspecies of the former were, in fact, separate species [69]. Indeed, it is feasible that the North American *Onchocerca* species complex, about which much is still unknown, could comprise undescribed *Onchocerca* diversity, similar to the pattern that we have witnessed in Asian suids and ungulates. Moreover, host–parasite biogeography appears to play a critical role in *Onchocerca* diversification. As noted by Uni et al. [69], *O. borneensis* and *O. dewittei* infect *Sus barbatus* Müller and *Sus scrofa vittatus* Boie in the Indomalayan region, but *O. japonica* and *O. dewittei* infect different subspecies of the same host species in the Palearctic and Indomalayan regions. Thus, when re-evaluating *Onchocerca* in the North American landscape, collecting specimens from sympatric and allopatric host ranges may yield more complete information about parasitic diversity.

## Conclusion

A cryptic *Onchocerca* species was found in Lake County, California, which is likely conspecific to isolates previously characterized from southern California. Putative vectors of this cryptic parasite include *S. tescorum* and *S. vittatum*. In addition, a previously unrecognized black fly vector, *S. clarum*, was discovered to be a potential vector. In order to understand the true biodiversity of the genus *Onchocerca* in North America, a complete continental re-evaluation of definitive hosts, vector associations, and geographic distribution is necessary through the integration of classical and molecular methods.

## Data Availability

The data supporting the conclusions of this article are included within the article. The sequences have been submitted to the GenBank database under the accession numbers MZ420192–MZ420198 (*nd5*).
